# Reply to Trollmann et al.: Perspective on LNP structure and simulation

**DOI:** 10.1073/pnas.2423433122

**Published:** 2025-02-24

**Authors:** Adiran Garaizar, David Díaz-Oviedo, Nina Zablowsky, Sami Rissanen, Johannes Köbberling, Jiawei Sun, Christoph Steiger, Patrick Steigemann, Florian A. Mann, Katharina Meier

**Affiliations:** ^a^Drug Discovery Sciences, Bayer Pharmaceuticals, Wuppertal 42113, Germany; ^b^Computational Life Science, Bayer Crop Science, Monheim am Rhein 40789, Germany; ^c^Lead Discovery, Nuvisan Innovation Campus Berlin, Berlin 13353, Germany; ^d^Chemical and Pharmaceutical Development, Bayer Pharmaceuticals, Turku 20210, Finland; ^e^Chemical and Pharmaceutical Development, Bayer Pharmaceuticals, Berlin 13353, Germany

We appreciate the interest of Trollmann et al. (1) and the opportunity for a scientific discussion on our recent manuscript (2).

Standard MARTINI simulations (3), including recent lipid nanoparticle (LNP) studies by MARTINI developers (4), use the Reaction Field method with a 1.1 nm electrostatic cut-off, implicitly screening interactions beyond this distance. For a charge density of 0.5 e/nm^3^, the average particle–particle spacing of 1.26 nm exceeds the cut-off, mitigating interactions and preventing extreme energies. Simulations of our system with Particle Mesh Ewald electrostatics and neutral cores showed similar ionizable lipid density profiles, with a minor change in density (<0.1 g/cm^3^) and hydration (<0.1 g/cm^3^). Ultimately, the 0.5 e/nm^3^ charge profiles refer to the LNP components and thus ions are not considered in the calculation of the profiles.

The radii of LNPs and their corresponding surface area-to-volume (SA/V) ratios are highly dependent on manufacturing parameters and might differ from their predicted thermodynamic equilibrium due to their metastable nature (5). The NPzT ensemble simulations aimed at replicating the experimental (SA/V) for LNPs from our experiments, yielding a 1,2-Dioctadecanoyl-sn-glycero-3-phosphocholin (DSPC) density of 0.75 molecules per nm^2^, consistent with prior experimental findings (6, 7). Corresponding Constant Number of Particles, Pressure and Temperature (NPT) simulations with comparable composition, employing a reaction field approach, resulted in the spontaneous self-assembly of a spherical LNP exhibiting analogous ionizable lipid densities. This self-assembly behavior is incompatible with extreme electrostatic repulsion and aligns with recent findings from MARTINI 3 simulations (4). NPT simulations with surface area coupling in a semi-isotropic ensemble lead to box width variations under 1 nm within our 10μs simulation window, noting that the tension at an LNP surface, given its metastability, may be uncertain. Ultimately, the simulations should align with the experimental conditions being modeled.

The extent of ionizable lipid relocation observed at low pH is a natural consequence of studying larger LNPs (100 to 200 nm), where the surface area per molecule is smaller than in previously studied systems (8, 9). This reduction limits surface accessibility, as ionizable lipids compete with cholesterol and DSPC (and polyethylene glycol) for fewer available sites, restricting their ability to relocate.

The authors are not aware of experimental evidence for conical ionizable lipids favoring bilayers at acidic pH. In contrast, numerous studies have documented the presence of inverse hexagonal arrangements at acidic pH (6, 10, 11). Furthermore, if RNA escaped from LNPs at neutral pH on the timescale of ns to μs (1, 8, 9), LNPs would be an especially unfavorable drug product and would not have achieved the success seen with mRNA vaccines during the COVID-19 pandemic.
